# Harnessing the Power of Next-Generation Sequencing in Wastewater-Based Epidemiology and Global Disease Surveillance

**DOI:** 10.1007/s12560-024-09616-0

**Published:** 2024-11-30

**Authors:** Kata Farkas, Rachel C. Williams, Luke S. Hillary, Alvaro Garcia-Delgado, Eleanor Jameson, Jessica L. Kevill, Matthew J. Wade, Jasmine M. S. Grimsley, Davey L. Jones

**Affiliations:** 1 School of Environmental and Natural Sciences, Bangor University, Bangor, Gwynedd LL57 2UW UK; 2 Verily Life Sciences LLC., South San Francisco, California 94080 USA; 3 Department of Plant Pathology, University of California Davis, Davis, California 95616 USA; 4 Data, Analytics & Surveillance Group, UK Health Security Agency, London, E14 4PU UK; 5 Moeseg Consulting, 64 Nile Street, London, N1 7SR England

**Keywords:** One Health, Pandemic preparedness, Viromics, Wastewater monitoring, Wastewater sequencing, Zoonotic diseases

## Abstract

Wastewater-based epidemiology (WBE) has emerged as a valuable surveillance tool for SARS-CoV-2 and other pathogens globally, providing insights into community-level infections, including asymptomatic and pre-symptomatic cases. While most WBE programmes focus on quantitative pathogen assessment, next-generation sequencing (NGS) approaches have enabled more detailed analyses, including variant and recombinant genotype identification for viruses like SARS-CoV-2 and poliovirus. Despite recent NGS advancements allowing for the detection of known and novel viruses in wastewater, many of these tools remain underutilised in routine WBE. This short review critically evaluates the applicability of common NGS tools in routine WBE programmes, assessing their capability for identifying emerging threats with epidemic or pandemic potential. Here, we provide evidence-based recommendations for integrating NGS techniques into WBE and the use of results for informed decision-making within a One Health framework, aiming to enhance global infectious disease surveillance and pandemic preparedness.

## Introduction

Wastewater-based epidemiology (WBE) has emerged as a critical tool in global infectious disease surveillance (e.g. SARS-CoV-2, poliovirus), offering unique insights into community health that complement clinical surveillance methods (Naughton et al., 2023). This approach enables rapid and quantitative detection of pathogens shed by humans in faeces and urine, with the data used to indicate infection levels within communities. WBE does not rely on infected individuals getting tested, which is especially important in situations with reduced healthcare access, or as for COVID-19, where many (32.4%) cases are asymptomatic (Shang et al., 2022). Furthermore, reports have shown that SARS-CoV-2 and its variants can be detected in wastewater up to 2 weeks before clinical detection, demonstrating the potential for its use as an early warning system (Jahn et al., 2022; Karthikeyan et al., 2022).

**Fig. 1:**
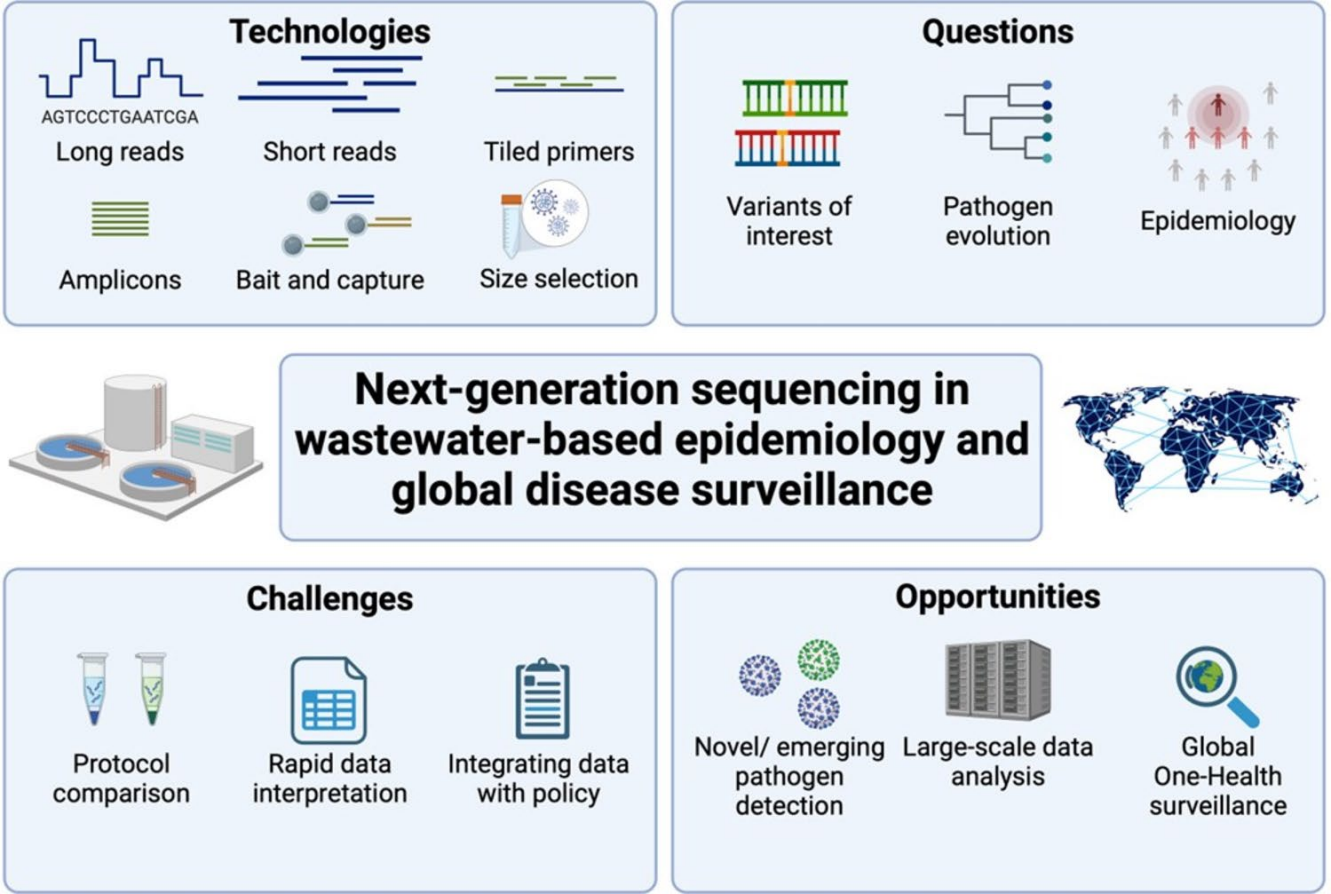
Summary of the sequencing-based approaches used for wastewater-based epidemiology.

The gold standard for quantitative virus detection in both clinical studies and WBE has been quantitative PCR (qPCR) (Ciannella et al., 2023). While qPCR has demonstrable value for detecting and quantifying SARS-CoV-2, its ability to identify different lineages and variants is limited. Unable to simultaneously target the increasing number of mutations constituting a variant, it cannot cover all mutations that may serve to assign a sample to the virus lineage. Further, redesign of qPCR oligos is required to identify novel variants, alongside assay validation before implementation. However, integrating next-generation sequencing (NGS) technologies with WBE has dramatically expanded our capacity to detect, characterise, and track pathogens at a community level, moving beyond simple quantification to detailed genomic analysis (Karthikeyan et al., 2022). While NGS provides WBE with a suite of tools for infectious disease tracking and assessment, the advantages and limitations of such approaches should be carefully considered. This short review critically evaluates the current landscape of NGS applications in WBE, examining their potential to transform global infectious disease surveillance and providing evidence-based recommendations for their integration into public health strategies (Fig. 1).

## Sequencing-Based Virus Identification Approaches in WBE

Viral genome sequencing provides detailed characterisation of mutations, allowing viral species and genotype identification, lineage classification, detection of variants of concern or interest (Iwamoto et al., 2023; Jahn et al., 2022; Karthikeyan et al., 2022), and phylogenetic analysis to trace the spatiotemporal spread of viruses (Nemudryi et al., 2020; Ni et al. 2021). This allows the monitoring of the emergence and transmission of pathogens with pandemic potential and reveals patterns of viral adaptation and host–pathogen interactions.

## Targeted Sequencing

Most frequently, sequencing approaches involve a primer-based targeted amplification of the viral genome. Primer-based methods (i.e. amplicon sequencing) use specific primers to amplify regions of viral genomes, which is often necessary for SARS-CoV-2 and other human pathogenic viruses (e.g. influenza, poliovirus) that can be found at very low concentrations in wastewater relative to gut-associated bacteriophages (e.g. crAssphage), which makes untargeted sequencing approaches impractical (Child et al., 2023). This allows for the detection and differentiation of viral genetic material even at low concentrations, while also reducing background noise from non-targeted sequences. For example, amplicon sequencing, which targets a ~600 base pair (bp) region of the SARS-CoV-2 genome, can distinguish variants at concentrations as low as 1–50 genome copies/µl (Ct values of 38) (Iwamoto et al., 2023). In targeted whole-genome amplification, multiple primer pairs are used to amplify overlapping sections of the full viral genome, allowing for more detailed phylogenetic analysis. However, the continuous evolution of SARS-CoV-2 and other viruses necessitates periodic redesign of primer panels to accommodate mutations at primer binding sites. This highlights a limitation of primer-based methods, as ongoing mutations may compromise detection if primers are not regularly updated.

Interpreting whole-genome sequencing data from wastewater samples presents unique challenges due to the complex mixture of genetic material from diverse sources. Unlike clinical samples, wastewater samples typically contain viral fragments shed by numerous individuals, potentially infected with different lineages of the same virus. This complexity necessitates the adoption of sophisticated data processing approaches to extract meaningful epidemiological information. Sequencing data have been used to produce consensus genome sequences for phylogenetic analyses (Nemudryi et al., 2020; Ni et al., 2021); this carries the risk of generating chimeric sequences that do not represent any actual viral genome, particularly in settings with high lineage diversity. Several tools have been developed to account for these complex mixtures (e.g. COJAC (Jahn et al., 2022), Freyja (Karthikeyan et al., 2022)), to identify different lineages in a sample, and to estimate their relative proportion. Combined with repeated sampling of representative sites, they can also identify infection trends of variants within a population.

By contrast to primer-based amplicon sequencing, probe-based approaches (e.g. hybrid capture) do not require amplification but instead use probes that bind to specific regions of the viral genome. These probe-based methods can capture larger genomic regions or multiple targets, including whole viral genomes, which allows for a more detailed phylogenetic analysis and accurate taxonomic classification (Nemudryi et al., 2020; Ni et al., 2021), although they typically require an order of magnitude higher viral concentrations (Ct values of 35) for robust detection (Amman et al., 2022). Commercial panels are often designed to enrich specific viral families or species of public health concern using probes that hybridise with target sequences. However, due to hybridisation conditions and probe design, these panels may also capture short conserved sequence motifs within larger viral genome fragments (Briese et al., 2015). This gives probe-based approaches viral discovery potential, enabling the detection of emerging viruses that share conserved genomic elements, even if they are not the primary targets of the panel (Sridhar et al., 2015). Although probe-based panels typically cover a limited number of viral families or species, this potential for viral discovery adds flexibility to their use in WBE. The choice of primers or probes depends on surveillance goals and the desired depth and breadth of genomic coverage.

The recently developed panel-based approaches to NGS detection of viral genomes enable sequencing of previously curated lists of pathogens. This panel-based approach enriches multiple targets (e.g. known pathogens) using baited capture. This method combines the efficiency of multiplex PCR combined with the insight gained from NGS, providing whole-genome sequencing data on a range of pathogens (Williams et al., 2024), yielding epidemiologically and clinically valuable sequence information (Graf et al., 2016). However, baited capture allows the detection of targets without the high read depth, tolerating greater divergence between target sequences (Rehn et al. 2021). It is theoretically capable of identifying a greater diversity of viruses using large panels of probes, but in practice, this can come as a trade-off for reduced sensitivity and specificity (Charre et al., 2020; Kapel et al., 2023).

Commercial panels focus on viruses posing a risk to public health and safety; they provide broad coverage of known pathogens and use reference genomes that are verified and used universally (Formenti et al., 2022). However, by function of their design, probes can only identify known viral targets, and users cannot discover new pathogens in circulation. However, hybrid capture can detect new mutations, but only for the baited target pathogens. Companies, such as Illumina and Twist Bioscience, are moving to remedy this drawback, with the commercial release of custom panel design providing a more flexible targeted approach. While not enabling true *de novo* pathogen discovery, these developments balance the need for standardised, broad-spectrum pathogen detection and the ability to adapt to emerging threats, but the development process can come at a substantial cost.

## Untargeted Viral Sequencing

Due to the lack of a universal marker gene, the untargeted sequencing of viruses remains challenging. However, advances in metagenomics and metatranscriptomics have enabled the sequencing and identification of unknown viral genomes in many ecosystems, including the human body, soil, wastewater, and aquatic environments (Lotze and Kost 2002). This offers a culture-independent approach to assess viral diversity, functions, and interaction with hosts and reservoir species, abundance, and evolution (Sommers et al. 2021). Furthermore, untargeted sequencing allows the identification and characterisation of emerging and novel viruses and provides critical knowledge that can be leveraged by infectious disease research and public health teams to develop more targeted sequencing methodologies.

There are many advantages to using untargeted sequencing, including the discovery of unknown viruses and emerging variants that escape detection by primer panels, an increased understanding of viral evolution, pathogenesis, and transmission, and aiding in the development of non-sequencing-based diagnostics. However, challenges arise due to many viruses belonging to genetically diverse groups, with mutation, recombination, and reassortment driving their evolution. This makes it challenging to assemble and annotate complete genomes from short sequencing reads for genomic characterisation. In addition, unlike cellular organisms, many viruses do not have double-stranded (ds)DNA genomes and the diversity of genetic material further complicates data analysis. Different sample preparation, enrichment, and virus identification strategies for dsDNA, single-stranded (ss)DNA, dsRNA, and ssRNA viruses should be applied, which can be time-consuming, expensive, and not directly comparable. Sequencing methodologies significantly impact viral detection and characterisation in wastewater samples, with each approach offering distinct advantages and limitations. Users can now choose between short-read, long-read, or length agnostic technology, adjusting the balance between coverage, depth, and error rates (Cook et al., 2024). The choice of sequencing strategy should be guided by specific surveillance goals, considering factors such as the need for broad virus discovery, routine monitoring of known pathogens, or comprehensive characterisation of dominant viruses, with a potential multi-pronged approach providing the most comprehensive view of the viral landscape in complex wastewater samples. Viral genomes can contain uncommon nucleotide sequences or variants that are difficult to detect with short-read technologies, especially in long or complex regions, making long-read sequencing technologies like Oxford Nanopore Technology (ONT) more suitable for capturing these features (Abebe et al., 2022; Amarasinghe et al., 2020).

The challenges of viral metagenomics (viromics) are further compounded by the sheer volume of previously unknown viruses that may co-infect any cellular species (e.g. bacteriophages, mycoviruses) or viral species (e.g. virophages, which co-infect alongside giant viruses, relying on their replication machinery) (Paez-Espino et al., 2019). Size-selective approaches that separate virus-like particles from cellular organisms and free nucleic acids (e.g. filtration, ultracentrifugation) enable the enrichment of viral genomes (Child et al., 2023). However, they may exclude giant viruses, viruses that clump or adhere, viruses sensitive to pressure, and proviruses integrated into the host’s genome (Ha et al., 2023). The purification of virus-like particles, extraction of viral nucleic acids, and preparation of RNA and DNA libraries are also technically challenging due to the complexity of sample matrices and the small sizes of viral genomes (Ramamurthy et al., 2017). Furthermore, the sheer volume and complexity of sequencing data require substantial computational resources and specialised expertise to fully exploit the rich information generated. Accurate and comprehensive assembly of viral genomes from mixed populations, especially for novel viruses, remains challenging. A significant proportion of sequences obtained in viromics studies cannot be assigned to known viral taxa, contributing to the "viral dark matter" (Santiago-Rodriguez and Hollister, 2022). Furthermore, distinguishing between clinically relevant viruses, the large volume of environmental background, and determining the host range of detected viruses, remain ongoing hurdles in wastewater viromics.

## NGS Platforms in Wastewater-Based Epidemiology

The choice of NGS platform is critical for the scope and quality of data obtained in WBE studies. The Illumina platform is widely used for its high read accuracy and sensitivity, making it particularly valuable for detecting low-abundance pathogens in complex wastewater samples. Its short-read sequencing offers higher throughput than other platforms using short-read sequencing technology, such as Thermo Fisher’s Ion Torrent. This allows for detailed quantification and variant detection from Illumina, essential for monitoring viral populations in wastewater (Karthikeyan et al., 2022). However, short-read lengths can limit the ability to resolve mutations co-occurring within a single genome, or to handle highly diverse viral populations that are often present in wastewater samples (Das et al., 2023). In contrast, ONT and Pacific Biosciences (PacBio) offer long-read sequencing, which is beneficial in overcoming these limitations by allowing the sequencing of entire viral genomes in a single read. This capability is particularly important for WBE, where viral genomes are often fragmented or mixed with genetic material from various hosts (Parra-Arroyo et al., 2023). ONT’s real-time sequencing capability and portability also enable more flexible and immediate field surveillance, offering significant advantages in outbreak monitoring and early warning systems. Despite these advantages, ONT’s slightly higher error rates compared to Illumina can pose challenges in base calling, which may require advanced error correction techniques (Długosz and Deorowicz, 2024; Safar et al., 2024). While here we cover the most widely used NGS platforms, such as Illumina, ONT, and PacBio, the list of available technologies is extensive and continually evolving, with each platform offering unique features and applications. The choices between available sequencing platforms should therefore be guided by specific surveillance objectives in WBE, whether it be high-accuracy pathogen quantification, tracking viral diversity, or real-time surveillance of emerging variants.

## Pandemic Preparedness and One Health

Viruses responsible for recent epidemics and pandemics have been zoonotic in origin (SARS, MERS, SARS-CoV-2, H1N1 and H5N1 influenza), transmitting from animals to humans in environments where WBE could be implemented, such as abattoirs, markets, and farm-derived sewage. This highlights the need for a One Health approach integrating human, animal, and environmental health surveillance to anticipate and manage zoonotic diseases. By implementing WBE with advanced sequencing techniques in strategic locations, such as areas with high human–animal interaction or potential zoonotic spillover sites, it is possible to create an early warning system that detects both known pathogens and novel viruses before they become major public health threats, thereby enhancing our global pandemic preparedness. The use of sequencing techniques in successfully monitoring SARS-CoV-2 during the pandemic highlights the potential of sequencing data to inform public health responses and enhance global pandemic preparedness. Real-time data obtained through these advanced methods enable timely interventions and help build a robust infrastructure for future disease surveillance.

## Utilisation of Sequencing in WBE Programmes

Many WBE programmes worldwide have incorporated sequencing into the monitoring of SARS-CoV-2 variants and for recombinant poliovirus surveillance (Iwamoto et al., 2023; Jahn et al., 2022; Karthikeyan et al., 2022; Klapsa et al., 2022). Targeted amplicon sequencing has been used for known viruses of interest due to the resource efficiency of sequencing specific targets. Identifying point mutations and recombinants promptly enables the early detection of novel variants and subtypes before clinical cases are noted, facilitating rapid public health responses (Iwamoto et al., 2023; Jahn et al., 2022). Implementing this approach for other viruses of global concern, e.g. influenza viruses, would decrease response time for public health safety.

Panel sequencing has typically been developed for clinical samples but has since been deployed for both broad and specific screening of wastewater (e.g. Ogunbayo et al., 2023; Williams et al., 2024). The role of wastewater surveillance in the COVID-19 pandemic and its potential to sample an entire community means that panel-based approaches are now being trialled for wastewater samples (Child et al., 2023; Williams et al., 2024). These preliminary studies suggest that the application of panel sequencing, targeting up to 3000 viruses in WBE, can be useful to get a snapshot of potentially harmful viruses. Therefore, it should be integrated as an occasional monitoring tool in WBE programmes, depending on emerging public health threats. However, this approach is relatively expensive and often fails to provide in-depth, strain-specific data or information on novel pathogens.

Due to the technical challenges and high cost of viromics approaches, few studies have been published on wastewater viromes. These have focused on the importance of methodological approaches (Child et al., 2023), the diversity of human RNA viruses in wastewater (Rothman et al., 2021), and the dispersal of human DNA and RNA viruses from wastewater to the aquatic environment (Adriaenssens et al., 2021). The studies demonstrated that viromics can identify novel and emerging human viruses in wastewater. However, applying viromics in WBE is still challenging due to the time and expertise needed for data analyses and the costs associated with sample preparation and sequencing.

To be effective, WBE requires the prompt processing and analysis of large numbers of samples for early detection of potential outbreaks in communities. Significant efforts have been made to automate workflows and move sample processing to wastewater treatment sites to save time and expense of sample transport (Hayase et al., 2023). While sample collection, concentration, RNA/DNA extraction, and PCR can now be automated and conducted in the field if necessary, library preparation and sequencing is more challenging. To date, only ONT sequencing (Nemudryi et al., 2020) offers portability, which is useful when considering on-site implementation and in low-resource settings. However, sequencing on ONT platforms has historically offered low quality data (Tshiabuila et al. 2022), masking single point mutations and requiring more sophisticated algorithms to align reads to reference genomes. Viral diversity and concentration in wastewater samples, combined with degradation during transport in the sewer network, create bioinformatic challenges not experienced with clinical samples. Optimisation of processing, extraction, and library preparation can reduce these challenges and enable routine field monitoring.

All forms of WBE sequencing generate large quantities of data in the mega-gigabyte range per sample, and the full exploitation of these data requires substantial computing resources. The global scale of WBE creates the opportunity for large meta-analyses of these datasets, which first require the open sharing of sequencing data through repositories such as the Sequencing Read Archive. While appropriate time-limited embargoes on public release protect the interest of data generators, standardised open access to sequencing data and associated metadata is essential to fully utilise these datasets.

Policies should address integrating WBE sequencing data with traditional clinical surveillance systems and define clear protocols for how this information should inform public health decision-making and emergency response procedures. WBE sequencing efforts should focus on strategic locations where new pathogens could emerge (e.g. large settlements, transport hubs, abattoirs) to support public health campaigns, vaccination programmes, and public health resource allocation. In addition, standardisation of sequencing methodologies and data reporting across different government organisations is crucial to enable meaningful comparisons and coordinated responses, necessitating the development of guidelines and best practices at national and international levels. Given the capital and operational cost of sequencing, national agencies should consider the initial investment in equipment and the ongoing costs of operation, data analysis, and workforce training. In the future, there may also be ethical issues that need to be considered when implementing high-resolution sequencing technologies.

## Conclusions and Implications

- Targeted sequencing enables the rapid (< 7 days) identification of variants in wastewater and other environmental samples, making it valuable for near real-time surveillance before health services are overwhelmed.
- Non-targeted viromics, while more costly and slower than amplicon-based approaches, and typically more expensive than probe-based methods due to the absence of targeted enrichment, are crucial for identifying novel and emerging pathogens, playing a vital role in pandemic preparedness.
- The integration of both targeted and non-targeted approaches in a tiered surveillance system can be deployed to provide comprehensive pathogen monitoring.
- Standardisation of sequencing methodologies and data reporting is essential for meaningful comparisons across regions and studies.
- A One Health approach, incorporating human, animal, and environmental health, is crucial for effective early warning systems and should guide the strategic implementation of WBE.
- Policy frameworks need to be developed to guide the integration of WBE sequencing data into public health decision-making processes.

## Data Availability

No datasets were generated or analysed during the current study.
